# A Liver Transplant Patient on Everolimus Treatment Presented with Acute Anterior Myocardial Infarction: Does the Type of Drug-eluting Stent Matter?

**DOI:** 10.4274/balkanmedj.galenos.2019.2019.12.123

**Published:** 2020-04-10

**Authors:** Muhammet Gürdoğan, Kenan Yalta, Mustafa Adem Yılmaztepe, Servet Altay, Ömer Ferudun Akkuş

**Affiliations:** 1 Department of Cardiology, Trakya University School of Medicine, Edirne, Turkey

To the Editor,

Liver transplantation (LT) is the most effective management strategy for end-stage liver disease and hepatocellular carcinoma (1).  Owing to the evolution of surgical techniques along with potent immunosuppressive agents and infection control, survival rates following LT have reached 90% and 80% at one and five years, respectively (1). In the general population, cardiovascular disease (CVD) constitutes the most common cause of adverse clinical outcomes in patients undergoing LT in the long-term (2). Primary percutaneous coronary intervention (PCI) is the recommended therapeutic strategy for ST-elevation myocardial infarction (STEMI) in post-LT patients (3). However, there exist no specific recommendations in the current literature regarding the most preferable type of drug-eluting stent (DES) during PCI in patients receiving long-term systemic immunosuppressive therapy.

A 55-year-old male patient was admitted to our clinic with chest pain. His electrocardiogram findings were consistent with an acute anterior STEMI. His history revealed chronic hepatitis B(+) and LT due to hepatocellular carcinoma three years earlier. Since his LT, the patient had been regularly receiving everolimus 0.75 mg (2 × 1), tenofovir 245 mg (1 × 1), ursodeoxycholic acid 250 mg (2 × 2), and esomeprazole 40 mg (1× 1). The initial hemogram, prothrombin time - international normalized ratio and liver and kidney function tests were all within normal limits. There was no contraindication to dual antiplatelet therapy or statins. Coronary angiography revealed a 99% thrombosed subtotal bifurcation lesion in the left anterior descending coronary artery along with insignificant atherosclerotic plaques in other coronary arteries. The culprit lesion was successfully managed with the culotte technique using two everolimus-coated stents (3.0 × 38 mm and 3.0 × 26 mm).

Currently, there have been only a couple of published case reports describing DES implantation following acute coronary syndrome in post-LT patients. Therefore, there are no specific recommendations regarding the impact of the presence and type of systemic immunosuppressive treatment in these patients on the choice of DES type to be implanted in this setting (4). Echeverri et al. previously reported the successful implantation of zotarolimus-coated stents in two patients with familial hypercholesterolemia, which was caused by STEMI and unstable angina pectoris, respectively). These patients had already undergone systemic immunosuppressive therapy with tacrolimus in the post-LT setting (5). However, in this case, we agreed on the implantation of an everolimus-coated coronary stent in our STEMI patient for whom everolimus had also been used as a systemic immunosuppressive agent since his LT, potentially considering the fact of that the additive impact of systemic everolimus (on top of its local release by the DES in a paracrine manner) might significantly contribute to the prevention of long-term stent restenosis in this setting. In the future, more transplant patients with successful surgical procedure outcomes are anticipated; therefore, longer survival rates under immunosuppressive therapy will be encountered in daily cardiology practices. Therefore, there is an obvious necessity for further studies that will shed light on particular DES preferences in patients receiving systemic immunosuppressive therapy.
